# Early Life-History Consequences of Growth-Hormone Transgenesis in Rainbow Trout Reared in Stream Ecosystem Mesocosms

**DOI:** 10.1371/journal.pone.0120173

**Published:** 2015-03-25

**Authors:** Glenn T. Crossin, L. Fredrik Sundström, Wendy E. Vandersteen, Robert H. Devlin

**Affiliations:** 1 Fisheries & Oceans Canada, Centre for Aquaculture and Environmental Research, West Vancouver Laboratory, 4160 Marine Drive, West Vancouver, British Columbia, V7V 1N6, Canada; 2 Department of Biology, Dalhousie University, Halifax, Nova Scotia, Canada B3H 4J1; 3 Uppsala University, Department of Ecology and Genetics/Animal Ecology, Norbyvägen 18 D, SE-752 36 Uppsala, Sweden; National University of Singapore, SINGAPORE

## Abstract

There is persistent commercial interest in the use of growth modified fishes for shortening production cycles and increasing overall food production, but there is concern over the potential impact that transgenic fishes might have if ever released into nature. To explore the ecological consequences of transgenic fish, we performed two experiments in which the early growth and survival of growth-hormone transgenic rainbow trout (*Oncorhynchus mykiss*) were assessed in naturalized stream mesocosms that either contained predators or were predator-free. We paid special attention to the survival bottleneck that occurs during the early life-history of salmonids, and conducted experiments at two age classes (first-feeding fry and 60 days post-first-feeding) that lie on either side of the bottleneck. In the late summer, the first-feeding transgenic trout could not match the growth potential of their wild-type siblings when reared in a hydrodynamically complex and oligotrophic environment, irrespective of predation pressure. Furthermore, overall survival of transgenic fry was lower than in wild-type (transgenic = 30% without predators, 8% with predators; wild-type = 81% without predators, 31% with predators). In the experiment with 60-day old fry, we explored the effects of the transgene in different genetic backgrounds (wild versus domesticated). We found no difference in overwinter survival but significantly higher growth by transgenic trout, irrespective of genetic background. We conclude that the high mortality of GH-transgenic trout during first-feeding reflects an inability to sustain the basic metabolic requirements necessary for life in complex, stream environments. However, when older, GH-transgenic fish display a competitive advantage over wild-type fry, and show greater growth and equal survival as wild-type. These results demonstrate how developmental age and time of year can influence the response of genotypes to environmental conditions. We therefore urge caution when extrapolating the results of GH-transgenesis risk assessment studies across multiple life-history or developmental stages.

## Introduction

There is persistent interest in the production of transgenic organisms for use in basic and applied bio-medical research, plant and animal agriculture, and aquaculture. Despite an appreciation for the potential ecological and evolutionary impacts that transgenic organisms might exact if ever released into nature (either intentionally or unintentionally), the pace at which empirical risk-assessment data have been generated has lagged behind advances in the technology [1]. This is complicated by the inherent difficulty in making ecologically realistic risk-assessments, with robust estimations of fitness parameters [2,3]. A main complication is that experimental release of fertile transgenic study animals into the wild is associated with unacceptably high levels of risk. Consequently, it is not possible to perform experiments in nature, yet data from such experiments are critically important when estimating ecological risk. Risk-assessment research must therefore depend on the use of contained laboratory mesocosm experiments that mirror, to the best extent possible, the physical and biological complexities found in nature. It is hoped that from such experiments fitness parameters might be estimated, thus allowing the ecological and evolutionary effects of transgenesis to be extrapolated to wild settings [1,2,3,4].

Domestication has long been the principal means for altering the growth of fishes, usually with the specific aim of increasing aquacultural production efficiency and yield, but there is large interest in the production of transgenic fishes to expedite this process [2]. To date, over 30 different species have been modified, many via growth-hormone (GH) transgenesis. Given that many aquacultural facilities operate in wild, open water areas, many researchers have performed experiments to examine ecological risks that transgenic fishes might pose should they escape from containment [2,4,5,6,7]. One common observation that these risk-assessment studies have found is that it is very difficult to predict the phenotype that a transgenic fish will display in a new environment, as phenotypes can be strongly influenced by environmental factors. For example, the extraordinary growth that GH over-expression supports in salmonid fishes in the laboratory (e.g. 37-fold increase in body size for coho salmon *Oncorhynchus kisutch* relative to their wild conspecifics; [8,9]), becomes muted when these same transgenic fishes are reared in semi-naturalized, heterogenous environments [10,11,12]. The reasons for this phenotypic plasticity are not fully known, but may arise from habitat structural complexity influencing fish behaviours, or different resource availability found in these naturalized environments (causing developmental and phenotypic plasticity). Certainly, phenotypic norms of reaction depend on the specific biotic and abiotic characteristics of the novel environment [10,13,14]. However, it is also likely that phenotypic reaction norms vary with life-history stage [15]. Thus, it is important to conduct species-specific risk assessments for GH-transgenic fishes, at multiple developmental life-history stages and environmental contexts, rather than generalizing limited experimental data to potential effects across species and environments.

In this paper, we present the first assessment of GH-transgenic rainbow trout (*Oncorhynchus mykiss*) growth and survival in naturalized mesocosm stream environments, and focus on their early life-history. Rainbow trout are studied widely in both ecological and bio-medical realms, and knowledge of their responses to GH-transgenesis would be valuable to each. The period of first-feeding, when larval fish transition from maternally-derived endogenous energy reserves to active foraging, is regarded a “critical period” in the life-history of salmonids, as these species inevitably pass through a mortality bottleneck [16,17]. The bulk of salmonid mortality occurs at this life-history stage (as high as 67% in Atlantic salmon, *Salmo salar*, [16,18]). In the simplest terms, food limitation can lead to competition and mortality, especially when the energetic demands of the environment exceed an individual’s ability to nutritional demands [17]. Food limitation results in hungry fish that take greater risks when searching for food, thus increasing susceptibility to predation [19]. Previous studies with coho and Atlantic salmon have shown that the risk-taking and mortality of GH-transgenic juveniles is increased relative to non-transgenic conspecifics, particularly when resources are scarce [6,20,21]. We therefore hypothesize that in early life-history stage, when larval trout begin active foraging, the metabolic scope of GH-transgenesis that enables extraordinary growth in laboratory settings when food is readily available would exert deleterious effects on rainbow trout survival when reared in complex, oligotrophic stream environments like those in which trout can be found naturally. Furthermore, the presence of natural predators would compound these effects. However, once fry survive the early stage of intense competition, we expect them to do better than wild-type owing to a stronger competitive ability as observed for GH transgenic coho salmon [11]. We also examined whether survival and growth of transgenic fish under these conditions are influenced by the genetic background into which the transgene introgress by crossing transgenic fish with domesticated fish.

Domestication has long been the principal means for artificially selecting desired phenotypes like enhanced growth, and many wild-type fish species respond with increased growth or yields as high as 7–10% per generation [21]. Although transgenesis and domestication are radically different in their approach (e.g. insertion of engineered promoter gene sequences versus multi-generational selective breeding programs), they nevertheless exploit many common physiological pathways to achieve a similar end [22,23]. Due to the high growth potential from both processes, there has been recent interest in producing transgenic animals from domesticated rather than wild stock. There is some evidence from laboratory studies that there is much variation in the extent to which transgenic/domesticated genomes affect growth phenotypes, depending on species and strain. Additive effects were seen in coho salmon [24], but only minimal effects were seen in rainbow trout [22]. Extending from the latter study, we predicted that when reared in naturalized stream environments the transgenic rainbow trout produced from domesticated stock would not differ in terms of growth or survival from transgenic fish produced from wild stock.

## Materials and Methods

Our research was approved by and conducted according to guidelines of the Department of Fisheries and Oceans Pacific Region Animal Care Committee (AUP 10–015). Experiments were conducted at Fisheries and Oceans Canada’s Centre for Aquaculture and Environmental Research located in West Vancouver, British Columbia, Canada. This federal government research facility has multiple containment screen systems to prevent the escape of genetically modified fish to the wild.

Transgenic genotypes of rainbow trout were produced via microinjection of a growth hormone gene construct (OnMTGH1) into eggs from wild Pennask Lake females [24]. The transgenic fish used in our experiments were produced by crossing multiple wild-caught Pennask Lake females to males hemizygous for the transgene construct, thus producing a population with equal proportions of offspring that were non-transgenic or were hemizygous transgenic (the latter henceforth called wild-type-transgenic or T_W_ fish). Wild-type fish (i.e. non-transgenic) were produced by crossing the same wild-caught females used to generate transgenic fish with wild-caught males. These wild-type offspring were thus half-siblings to the transgenic offspring and are henceforth called W-fish.

Domesticated rainbow trout (D_W_) originated from a wild genetic stock and were obtained from the Campbell Lake trout farm, Little Fort, BC. Domestic-transgenic-fish (T_D/W_) were produced by crossing the same transgenic rainbow trout strain described above (with wild genetic background) to domesticated females (D_W_), thereby producing a 50:50 ratio (D_W_ to T_D/W_) transgenic and non-transgenic fish that both were interstrain hybrid with domestic/wild genetic backgrounds.

### Experimental mesocosms

**Fig 1:**
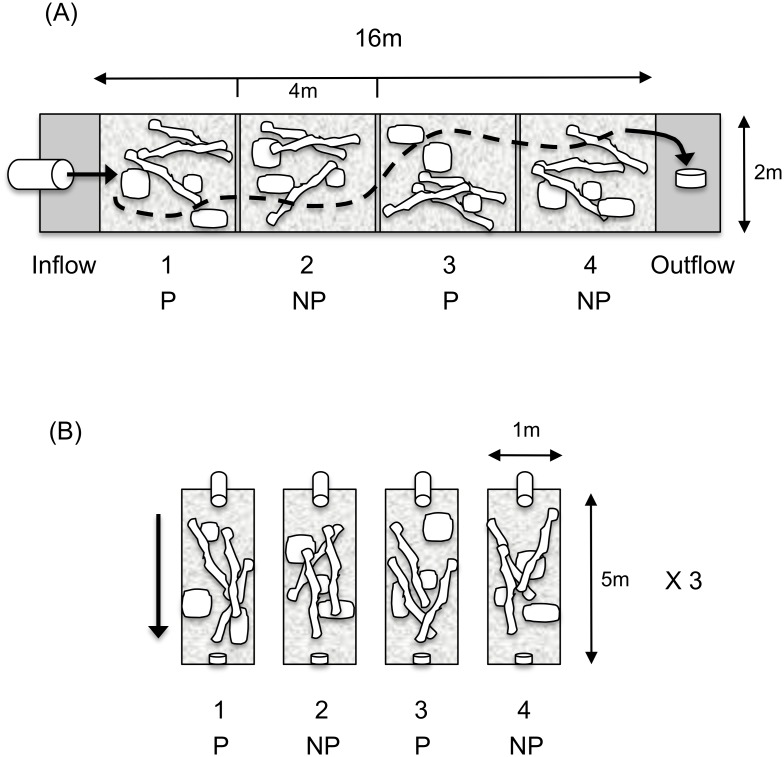
Design of the mesocosm stream tanks. Experiment 1 was conducted in a 16 metre re-circulating stream tank (panel A), landscaped with stream gravels, woody debris, and small boulders (indicated by shapes). This landscaping resulted in a main channel current that ran the length of the mesocosm (indicated by the dashed line), creating riffles and glides, away from which pools and back-eddies formed amid the woody/cobbly structures. Experiment 2 was conducted in twelve of replicated 5 metre mesocosm tanks (panel B), landscaped in a similar manner as the mesocosm stream in experiment 1. See Material and methods section and Table 1 for additional details.

We used two different stream mesocosm configurations in our experiments, which varied by size (each are described below). Irrespective of experiment, all mesocosms were landscaped in the same manner. Each contained coarse stream gravels and were supplied with surface water collected directly from Cypress Creek, which is a natural stream running adjacent to the laboratory facility and in which trout and salmon spawn naturally. This unfiltered creek water contained live invertebrates and protists, at a biomass characterizing it as naturally oligotrophic. Water in the stream tanks was maintained via both flow-through and recirculation techniques. Outflows in each stream were maintained at a rate of 10 Lmin^-1^. Due to gravel contouring, water depth varied from 10–45 cm, within each stream. This contouring created fast riffle and slower glide areas within each stream. Streams were also landscaped with submerged tree trunks, limbs, branches, small rocks and boulders, live vegetation, and leaf litter, thus adding greater hydrodynamic complexity and stream authenticity. A central current ran the length of each stream (see Fig. 1), and woody debris and cobble allowed many still pools and back eddies to form away from the main channel and riffle areas. All mesocosm streams were housed in a contained facility with a transparent, semi-permeable roof, which exposed them to natural daily photoperiods and ambient environmental temperatures.

**Table 1:** Initial sizes of wild-type, domestic/wild hybrid, and transgenic rainbow trout used in experiments, and the total numbers of fish used in each.

Mesocosm experiment	Genotype	Length (mm) ± SEM	Mass (g) ± SEM	*N* used for initial size estimates	*N* used in experiment
First feeding	W	23 ± 0.12	0.12 ± 0.02	51	400
	T_W_	22 ± 0.11	0.11 ± 0.01	49	400
	W control	23 ± 0.12	0.12 ± 0.02	100	150
	T_W_ control	22 ± 0.11	0.11 ± 0.01	100	100
60 day post	W	41 ± 0.87	0.62 ± 0.03	19	322
first feeding	T_W_	50 ± 1.51	1.25 ± 0.13	21	270
	D_W_	43 ± 1.30	0.85 ± 0.08	13	290
	T_D/W_	50 ± 1.16	1.36 ± 0.10	26	301

Initial mean sizes of mesocosm stream fish were calculated from a subsample of each family and genotype. For the control fish in mesocosm stream experiment 1, there were no statistically significant differences in lengths or masses between genotypes. Numbers of fish used in each experiment are also presented. Note that within each mesocosm stream experiment, genotypes were exposed to different experimental treatments (e.g. with predators, without predators). See the Material and Methods section and Fig. 1 for further details.

W = wild-type.

T_W_ = transgenic from wild background.

D_W_ = domestic/wild hybrid.

T_D/W_ = transgenic from domestic/wild hybrid background.

Control = fish held in environmentally stable hatchery tanks, fed maximally, and without predation pressure or complex flow regimes.

### Mesocosm experiment 1: first-feeding

In the first experiment, a single mesocosm stream tank was used, measuring 16 m × 2 m × 1 m (see Fig. 1A). Four replicate stream sections (4 m × 2 m × 1 m) were established by the placement of mesh screens at evenly spaced intervals.

We used rainbow trout fry that had just absorbed their yolk masses and were beginning to forage for the first time. The experiment was conducted over a 25 day period in summer from 13 August to 5 September 2010, which parallels the natural phenology of the wild Pennask Lake trout population from which they are derived.

On day 1 of the experiment, 100 first-feeding fry were taken directly from hatching trays (13 August 2010). They were measured (length [mm] and mass [g]) and adipose fin clipped for genotyping using a Q-PCR test specific for the GH transgene [9]. The proportion of transgenic to wild-type fish from this sample was approximately even (49 T_W_, 51 W), so we assumed an even distribution of genotypes in the larger pool of offspring still in the hatching trays. These fin-clipped fry were not used in mesocosm stream experiments.

An additional 200 fry were taken from hatching trays and were divided into two 170L hatchery tanks. We monitored the growth and survival of these “control” fry to that of the fry used in our experiment (detailed below). These hatchery fry were maintained in a predator free environment, without food limitation or complex hydrodynamics. They thus served as a point of comparison with the fry reared simultaneously in the stream mesocosm experiment. Mean lengths and masses of these control fry also served as day 1 population references for length and mass (see Table 1). Control fry were fed live brine shrimp daily at 0.5% of total biomass, and survival was also monitored daily. Mortalities were collected daily and fin clipped for genotyping via Q-PCR.

As the initial hatchery sample (see above) showed a near-even proportion of T_W_ to W, we released 200 sibling fry to each of 4 replicate stream segments (for a total 800 fry), assuming that each stream segment would then contain 100 transgenic and 100 wild-type fry. Here we could monitor the potential for differential growth and survival between transgenic and wild-type fish under semi-natural environmental conditions. For the first 14 days after their introduction to the stream mesocosm, fry were allowed to acclimate to their new, complex stream environment without yet being exposed to predators. During this period, fry could forage freely on the invertebrates and protists present in the Cypress Creek water, but live brine shrimp (*Artemia* spp.) were added daily as supplemental feed. Shrimp were provided at 0.5% of total fry biomass, which we classified as oligotrophic-like conditions (but less so relative to [6]). Qualitative assessments of water quality were made daily, as were visual assessments of fry survival; any fish that died during this time would collect on the mesh at the downstream end of the segment. These were fin clipped and genotyped.

On day 14, a single, wild coastal cutthroat trout (*O*. *clarkii clarkii*) was introduced into 2 of the 4 stream segments (segments 1 and 3; Fig. 1A). These yearling predators averaged 18 cm ± 0.23 SEM in length. One each day thereafter, we examined the stream segments for dead fry. If found (always pressed against the downstream screen), they were collected for genotyping. On day 24, the experiment was terminated, and all stream structure and predators were carefully removed from the individual segments. All surviving rainbow trout fry were captured, measured, weighed, adipose fin-clipped. Fin clips from all mortalities (collected during the pre-predator acclimation period), and all survivors (at the end of the experiment) were euthanized with an overdose of tricaine mesylate (e.g. MS-222) and genotyped.

We should note that 2 days before and 2 days after the introduction of predators on day 14, we made careful visual assessments of fry in each stream segment, and quantified the number of fish seen. Fish that were not visible were presumably hiding within the stream gravel, behind or beneath boulders, or in dense woody debris areas, or (in segments with predators) had been consumed.

### Mesocosm experiment 2: 60 days beyond first-feeding

Rainbow trout were reared in predator-free hatchery tanks (170L) and fed satiating daily ration for 60 days beyond first feeding to assure that fish partaking in this experiment had survived the sensitive early period. Next, (day 1 of the experiment; 9 October 2007), 100 rainbow trout fry were transferred to each of 12 experimental stream mesocosm tanks (5 m × 1 m × 0.5 m and landscaped in an identical manner as above; [10]). In each of six tanks, there was a 50:50 mix of non-transgenic and transgenic fry with a wild genetic background, and in the other six there was a 50:50 mix of non-transgenic and transgenic fry both with a wild/domestic hybrid genetic background (Fig. 1B). Exact numbers of each genotype going into each tank were determined by Q-PCR analysis of adipose fin clips.

Fry were fed once daily on brine shrimp at ca. 1% of fry biomass per tank (i.e. food abundance was higher than in experiment 1), and once-weekly supplemented with roughly 50 black benthic annelids (*Tubifex* sp.) and 25 surface-drifting crickets (*Acheta domestica*, 2–3 mm) per tank. In half of the tanks one older rainbow trout (average 490 g, 320 mm) and two cutthroat trout (average 27 g, 140 mm) were introduced as predators at day 18 and remained until the end of the experiment. At the end of the experiment, on day 132 (18 February 2008), predators were removed, and all fry were captured, euthanized, weighed, measured, fin-clipped, and genotyped.

### Statistical analyses

In experiment 1, we compared the initial sizes (length, mass) of T_W_ and W fry in the hatchery control sample using analysis of covariance (ANCOVA), where body mass was compared between genotypes while controlling for allometry, with body length as the covariate. The survival of fry in the stream mesocosm was assessed at two times: during the acclimation period (pre-predators, in experiment 1 only), and again at the end of the experiment (post-predators, in experiments 1 and 2). For each period, survival was compared by treatment, and genotype nested within treatment (nested ANOVA).

For all surviving fish in experiments 1 and 2, we calculated specific growth rate in mass (SGM) and in length (SGL) during the experiment. Growth rates were examined with a similar nested ANOVA model as above. SGM was defined as 100 · (lnW_1_—lnW_0_) / t, where W_0_ and W_1_ are the weights at the start (pooled estimate) and end (individual measures) of the experiment, and t is the time in days that elapsed between the two periods. SGL was estimated using the same equation but with length substituting mass. In experiment 1, the survival of T_W_ and W fry in the hatchery control tanks was used as a point of comparison for the survival of mesocosm stream fry. The survival and growth of control fry were analyzed separately from the mesocosm stream fry since they were reference fish and not true controls. Growth and survival for the reference fish was compared between genotypes using t-tests.

Values presented in all figures represent means +SEM, with statistical contrasts via Tukey’s tests.

## Results

### Mesocosm experiment 1: first feeding

At the beginning of this experiment, there was no genotypic difference in the relationship between initial mass and length of fry; body mass did not differ between genotypes, despite a significant co-varying effect of body length (ANCOVA full model *F*_3,39_ = 63.84, *P*<0.001; genotype *F* = 0.418, *P* = 0.521; length covariate *F* = 182.4, *P*<0.001; genotype*length interaction *F* = 1.674, *P* = 0.202). See Table 1 for a summary of first-feeding lengths and masses. All fry thus entered the mesocosm stream at the same age and similar lengths and masses.

**Fig 2:**
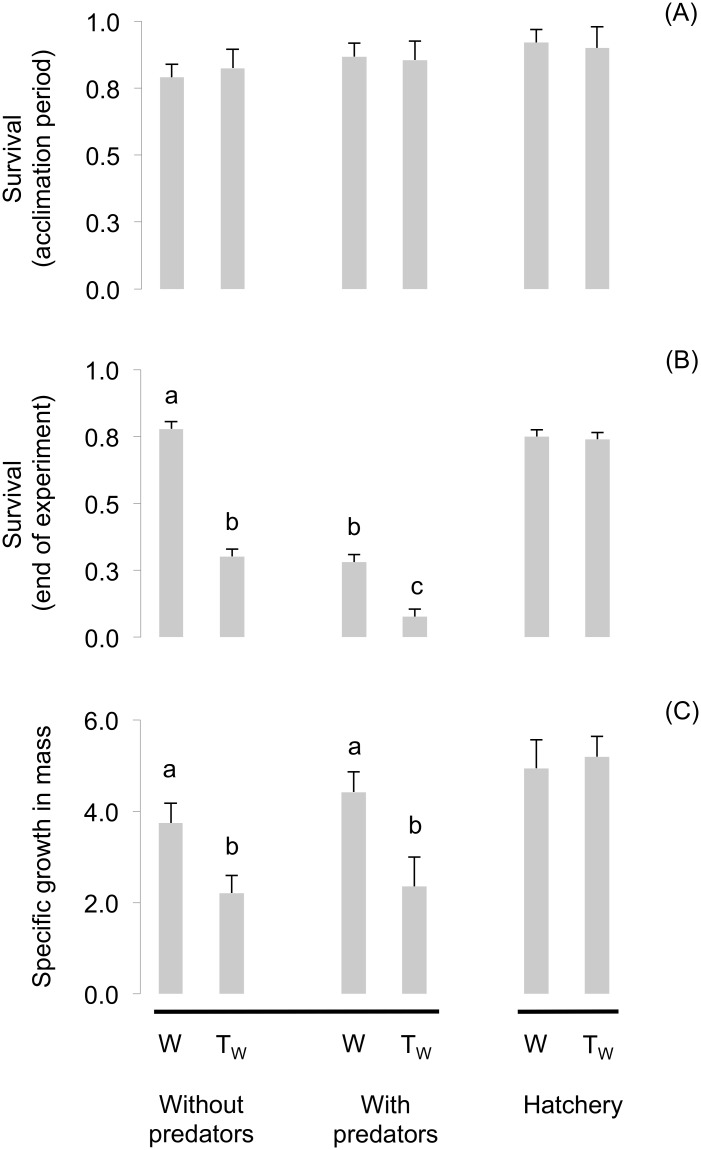
Survival and growth of wild-type (W) and transgenic-wild-type (T_W_) rainbow trout in a naturalized mesocosm stream environment. Survival was assessed for recently hatched trout at the end of the first two weeks of stream life, just prior to the introduction of predators to the system (Panel A), and again at the end of the experiment, after predators were added to some treatment sections (Panel B). Specific growth in mass was measured in all surviving fish at the end of the experiment (Panel C). Mesocosm stream fish were compared via a nested ANOVA model, with treatment and genotype] as main effects. Due to different experimental setting and design, control fish were analyzed separately but are presented here for comparison. Bars are least square means ± SEM. Different letters indicate statistically significant contrasts when = 0.05.

**Fig 3:**
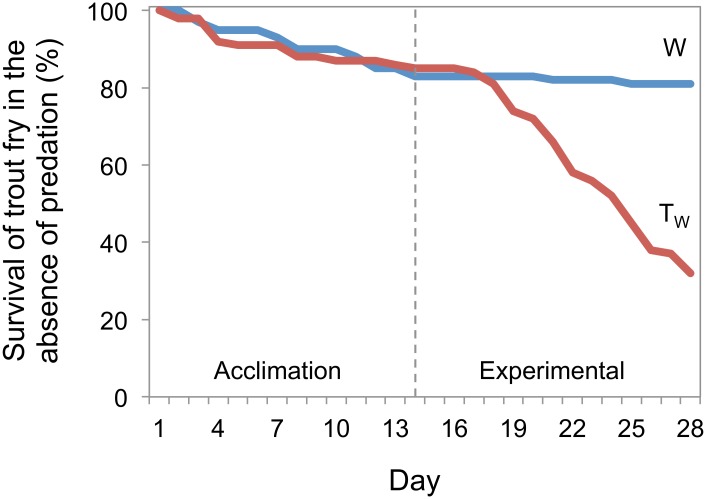
Comparison of daily survival of wild-type (W) and transgenic-wild-type (T_W_) rainbow trout in the predator-free stream segments only, throughout the entire duration of the mesocosm experiment.

During the 14-day acclimation period, before predators were introduced to the mesocosm, there were no genotypic differences in fry survival (ANOVA, *F*_3,11_ = 0.449, *P* = 0.725) (Fig. 2A). However, survival until the end of the experiment differed significantly depending on the presence or absence of predators, but irrespective of predator treatment survival was higher in W fry (81% non-predator, 31% predator) than in T_W_ fry (32% non-predator, 8% predator) (full model *F*_3,11_ = 87.538, *P*<0.001; predator treatment *F* = 100.545, *P*<0.001; genotype[treatment] *F* = 59.233, *P*<0.001) (Fig. 2B). In the “no predator” stream treatments, T_W_ survival was lower than W survival (T_W_ = 0.32 ± 0.04 SEM, W = 0.81 ± 0.03 SEM) (Figs. 2B and 3), and the same pattern was observed in the “predator” treatments (T_W_ = 0.08 ± 0.04 SEM, W = 0.31 ± 0.03 SEM). In contrast, survival of control fry in the hatchery tanks (simple environment, no predators) did not differ between genotypes, either during the pre-predator acclimation period (T_W_ = 0.96 ± 0.02 SEM, W = 0.95 ± 0.02 SEM; t-test *F*_1,3_ = 0.059, *P* = 0.831), or at the end of the experiment (T_W_ = 0.74 ± 0.02 SEM, W = 0.75 ± 0.02 SEM; t-test *F*_1,3_ = 0.020, *P* = 0.900) (Fig. 2A and B).

Regarding growth, there were significant SGM differences in the mesocosm stream fish, with lower growth in T_W_ versus W fish in both treatment (nested ANOVA *F*_3,11_ = 7.850, *P* = 0.038; treatment *F* = 1.157, *P* = 0.343, genotype[treatment] *F* = 11.197, *P* = 0.023) (Fig. 2C). There were no differences in SGL between treatments and genotypes (*F*_3,11_ = 4.850, *P* = 0.238). Growth of W mesocosm stream fish from both treatment groups was similar to those of the hatchery control fry (Fig. 2C); in the hatchery, SGM was similar between T_W_ and W control fry, although this difference was marginally non-significant (t-test *F*_1,3_ = 10.413, *P* = 0.084) (Fig. 2C). Although not presented in Fig. 2, SGL of control fry was not significantly different between genotypes (t-test *F*_1,3_ = 5.923, *P* = 0.135) (Fig. 2C).

To summarize the survival of rainbow trout throughout the duration of the mesocosm experiment, Fig. 3 presents survival curves for T_W_ and W fish in the predator-free stream segments only, as dead trout were not consumed in these segments, and therefore recoverable at the end of each day.

**Fig 4:**
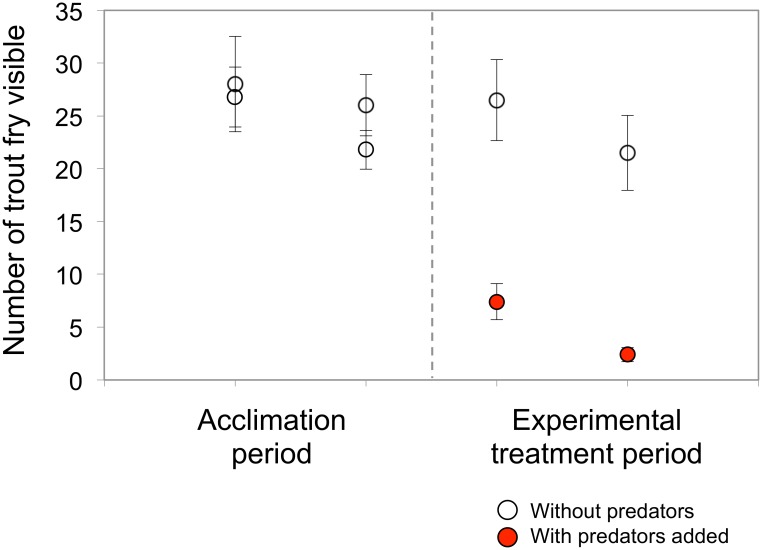
The number of rainbow trout visible in each stream segment was determined at two times: daily, during the first two weeks prior to the introduction of predators (left panel), and once, just two days after predators were added to half of the stream segments (right panel). An initial total of 200 rainbow trout fry (100 W, 100 T_W_) were placed in each stream segment for a total 800 fry. White circles signify mean numbers of fish visible in the predator-free stream segments, while black circles signify means from the predator-treated segments. The reduced number of fish visible when predators were present (black circles) are statistically significant departures from pre-predator numbers in the left panel (repeated measures, *P* = 0.003).

From visual observations of stream segments during the course of the experiment, fewer trout fry were seen in sections containing predators compared to those without predators, suggesting that fry hid when the threat of predation was high (Fig. 4; genotypes unknown; repeated measures ANOVA, exact *F*_1,6_ = 24.640, *P* = 0.003).

Among surviving fish from both the mesocosm experiment and the hatchery control tanks, genotypes did not differ in mean body length (W = 37 mm 0.03 SEM, T_W_ = 37 mm 0.01, W_control_ = 35 mm ± 0.39, T_Wcontrol_ = 36 mm 0.41).

### Mesocosm experiment 2: 60 days beyond first feeding

**Fig 5:**
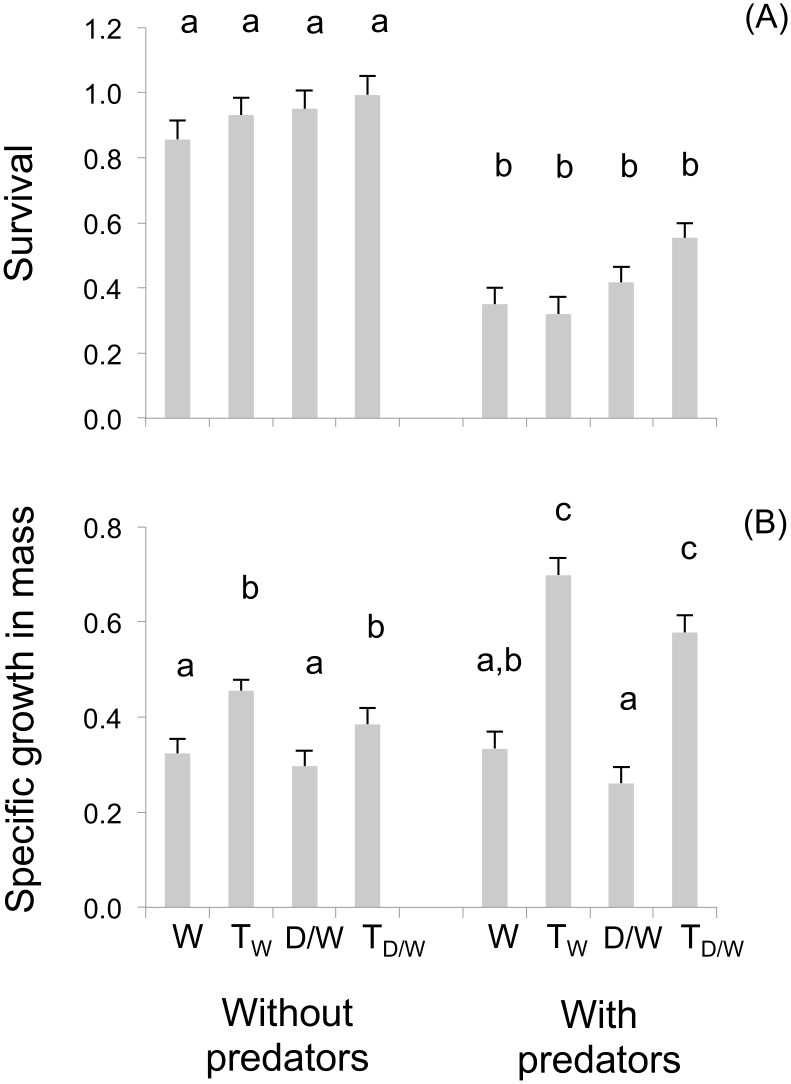
Survival and growth of wild-type (W), transgenic-wild-type (T_W_), domesticated/wild non-transgenic (D_W_), and transgenic-domestic/wild (T_D/W_) rainbow trout in naturalized stream tank environments with and without predators (Panel A). Specific growth in mass was measured in all surviving fish at the end of the experiment (Panel B). Bars are least square means ± SEM. Different letters indicate statistically significant contrasts when = 0.05.

After 60 days of feeding in the hatchery, the rainbow trout fry going into this experiment differed significantly in size among genotypes (Table 1). At the end of the 132 day mesocosm experiment, survival in the predator-free stream tanks did not differ by genotype (D_W_ vs. T_W_), and was overall very high at 86–99% (Fig. 5A). However, survival was significantly reduced in the predator stream tanks (*F*_7,16_ = 24.891, *P* < 0.001) to 35–55%, but without differences among genotypes (see Tukey’s Test contrasts in Fig. 5A). With respect to specific growth in mass, transgenic fry from both backgrounds (T_W_ and T_D/W_) had significantly higher final growth than the non-transgenic W and D_W_ fish, and this difference occurred in stream tanks with and without predators (*F*_7,16_ = 22.310, *P* < 0.001; see contrasts in Fig. 5B). In no case did hybridization with a domesticated genetic background enhance the growth of transgenic or non-transgenic fry relative to a wild genetic background.

## Discussion

### Survival effects of transgenic fish in a naturalized stream mesocosm

The principal aim of this work was to examine the early life-history consequence of GH transgenesis on the behaviour, growth, and survival of rainbow trout. The mesocosm stream experiments were designed to simulate the complex, oligotrophic stream conditions common to regions where rainbow trout have evolved (i.e. western North America). The results of our first experiment show that when reared in a naturalized stream mesocosm environment, transgenic trout fry were more susceptible to predation than their wild-type half-siblings (Fig. 2), which is a result consistent with other studies of transgenic fishes [6,20] and which supports our main prediction. However, even in streams that did not have predators, transgenic fry still suffered significantly higher mortality relative to the wild-type (Fig. 3). The growth data showed a similar result, with transgenic fish incapable of matching the growth of their wild-type siblings, whether predators were present or not. Lower growth and survival are similar to that seen in studies of first-feeding transgenic coho salmon [6,11], and in Atlantic salmon where a greater loss in weight was observed for transgenic vs. wild-type animals [12] under conditions of very low food availability. Interestingly, the transgenic and wild-type control fish reared in the hatchery showed equal growth and survival during the experimental period, which suggests that the GH transgene was not yet active enough at this early life-history stage to cause major changes in growth, but active enough to exert pleiotropic effects on food-seeking behaviour and survival (Fig. 2B-C). Moreau et al. [25] also did not find growth enhancement at early stages in GH transgenic Atlantic salmon, which is distinct from that seen with several strains of coho salmon that show developmental advancement even in the larval stage, and in some cases lower body size in transgenic individuals prior to first feeding [14,26,27]. Future analyses of GH transgene expression during this period of early stream life in rainbow trout would do much to reveal the connections between GH and food-seeking behavior, even in the absence of detectable growth enhancement.

The observed growth and survival effects are striking because they highlight how transgenesis can impose a significant fitness cost at an early life-history stage of salmonids when selection pressure is naturally high [16]. The mechanisms driving the reduced survival of transgenic trout are presumably linked in part to a loss of behavioural plasticity that results in risky foraging behaviours in the presence of predators, thereby increasing susceptibility to predation. However, as our results show, survival of transgenic trout was reduced even in the absence of predators, which probably reflects an inability to satiate their greater metabolic needs when reared in a complex but food-limited environment. Indeed, the transgenic trout that survived both the predator- and predator-free treatments had significantly lower growth rates than their counterparts in the controlled, predator-free hatchery environment. This suggests that transgenic trout with high growth rates were selectively removed from the population via the combined influence of resource constraint and predation [28]. Although many studies that have demonstrated how fast growth during early life can positively influence many subsequent biological processes, including foraging success and competitive ability [29], the timing of seaward migration [30], osmoregulatory physiology [31], and reproductive success [32], fast growth does not necessarily mean ‘maximal’ possible growth, and in salmonids natural growth rates have not evolved to be maximal (as evidenced by successful artificial selection for rapid growth in fish farms; e.g. [33]). Theoretically, natural selection allows for the existence of a range of optimal, sub-maximal growth rates in salmonids, beyond which deleterious effects on survival occur due to a range of possible trade-offs [34]. In wild fish there is ample evidence of negative fitness effects of very fast growth [19,35], and studies of transgenic and domesticated fish, which are forced towards maximal growth, show additional costs [6,20,36,37,38,39,40]. Our results corroborate these ideas; transgenic trout seem ill-suited for growth and survival reared in a simulated wild environment.

### Effects of transgenesis on a hybrid wild/domestic genetic background

The second aim of our study was to examine the early life-history effects of transgenesis in wild-type versus wild-domesticated hybrid genetic backgrounds. The results of experiment 2 show that GH transgenesis confers no discernable survival advantage to trout, at least in winter which is when this experiment was run; transgenic and non-transgenic fish from both W and D_W_ backgrounds survived equally, irrespective of predator treatment. In terms of growth however, there were differences, with both T_W_ and T_DW_ growing at faster rates than their non-transgenic siblings. We suggest three reasons for this growth difference. First, experiment 2 was conducted in winter, when the growth of transgenic fish was maintained whereas it is usually slowed or suspended in wild fish [6,14,41]. Second, experiment 2 was conducted using trout fry that were not first-feeding; trout were 60 days old at the start of the experiment and presumably past the critical mortality bottleneck period (i.e. 2–3 weeks after emergence; [16]). A study by Sundström et al. [38] showed that when transgenic salmonids are exposed to predators after this vulnerable period of emergence, selection for higher growth rates can result. Third, because of this age difference, there were already significant departures in body size among transgenic and non-transgenic fish at the start of the experiment, the former being 20% larger, which indicates that the transgene was already active and fostering higher growth rates. In combination with experiment 1, this indicates that the transgene activates soon after the first month post first feeding.

Despite the growth differences between transgenic and non-transgenic fish, there was no apparent growth advantage of GH transgenesis when imposed over an already fast-growing domesticated genetic background [24]. This suggests that the metabolic pathways exploited by transgenesis are similar to those exploited through domestication, and that no additive effect on growth occurs [22,23]. Interestingly though, the non-transgenic domesticated fish did not differ in growth from the non-transgenic wild-type fish, despite the fact that D_W_ fish can outgrow W fish in controlled hatchery environments free from winter and predator effects [42]. This suggests that D_W_ fish may also suspend growth in winter like the W fish do, or that growth is limited by competitive interference by the larger transgenic fish in oligotrophic stream environments [6,12,14].

### Conclusion

To place our results in a larger context, our study suggests that if the present strain of GH-transgenic trout ever found its way into nature, its success would likely be constrained by the interacting effects of low resource availability and high predation risk. In our experiments, we sought to replicate the naturally oligotrophic stream conditions and predation regimes that rainbow trout experience in western North America. While we feel that our results have lent new insights to the potential ecological and fitness consequences of GH transgenesis, attempts to extend these to risk assessment should be made with caution. First, although native to western North America, rainbow trout have been introduced and stocked all over the world, and now occupy habitats which range from oligotrophic to eutrophic. Future studies should therefore examine the fitness effects of transgenesis under a range of different food conditions, using similar mesocosm type experiments described here and elsewhere. Second, as life-time fitness is a composite of survival estimates made at many different developmental and life-history stages, and in a complex array of environments, our results suggest that transgenic fry born in the wild would be subjected to a narrow survival bottleneck (experiment 1) and be preferentially eliminated relative to wild types. However, those surviving this bottleneck may be able to outgrow wild types (experiment 2). Thus, controlled studies of survival at additional life stages, as well as studies of reproductive success would be required for a comprehensive assessment of life-time fitness, transgenic frequencies through time, and any associated ecological impacts [1]. Third, our study was conducted with only one transgenic line (family). There remains a small, although probably unlikely, possibility that the introduced GH transgene was integrated at a specific genomic region in our fish which disrupted the function of an essential gene. Future studies would benefit by examining multiple transgenic lines before generalizing the survival and growth effects of GH overexpression observed here.

Finally, while the metabolic effects of transgenesis and domestication appear to be similar, the multi-generational consequences of transgene vs. domesticated genome introgression are expected to be quite different. In fact, research has shown that the phenotypic effects of transgenesis remain constant with each backcross to wild type, whereas introgression of a domesticated genotype diminishes with each backcross generation [42,43,44,45]. Risk assessments should therefore be cognizant of these different effects, especially when assessing the potential for multigenerational risk of escaped or released fish to wild breeding populations [42,43,44,45,46,47,48].
